# Postnatal nutrition environment reprograms renal DNA methylation patterns in offspring of maternal protein-restricted stroke-prone spontaneously hypertensive rats

**DOI:** 10.3389/fnut.2023.1134955

**Published:** 2023-04-13

**Authors:** Chika Ando, Sihui Ma, Moe Miyoshi, Kyohei Furukawa, Xuguang Li, Huijuan Jia, Hisanori Kato

**Affiliations:** ^1^Health Nutrition, Graduate School of Agricultural and Life Sciences, The University of Tokyo, Tokyo, Japan; ^2^Faculty of Sport Sciences, Waseda University, Tokorozawa, Japan; ^3^Animal Nutrition, Life Sciences, Graduate School of Agricultural Science, Tohoku University, Sendai, Japan

**Keywords:** DNA methylation, epigenetics, gene expression, hypertension, kidney, maternal protein restriction, postnatal nutritional environments

## Abstract

Maternal malnutrition hampers the offspring health by manipulating the epigenome. Recent studies indicate that the changes in DNA methylation could be reversed by afterbirth nutrition supplementation. In this study, we used DNA methylation arrays to comprehensively investigate the DNA methylation status of the renal promoter regions and the effects of postnatal protein intake on DNA methylation. We fed stroke-prone spontaneously hypertensive (SHRSP) rat dams a normal diet or a low-protein diet during pregnancy, and their 4-week-old male offspring were fed a normal diet or a high−/low-protein diet for 2 weeks. We found that the methylation status of 2,395 differentially methylated DNA regions was reprogrammed, and 34 genes were reset by different levels of postnatal protein intake in the offspring. Among these genes, *Adora2b*, *Trpc5*, *Ar*, *Xrcc2*, and *Atp1b1* are involved in renal disease and blood pressure regulation. Our findings indicate that postnatal nutritional interventions can potentially reprogram epigenetic changes, providing novel therapeutic and preventive epigenetic targets for salt-sensitive hypertension.

## 1. Introduction

Hypertension is a serious medical condition that significantly increases the risks of cardiovascular, brain, renal, and other diseases, and is a major cause of premature death worldwide (1). Although the pathogenesis of hypertension is not yet fully elucidated, current evidence indicates that the origins of hypertension can be linked to the very early life stage. The developmental origins of health and disease (DOHaD) concept, which considers postnatal exposures to environmental stimuli as important risk factors for non-communicable diseases (2), has increasingly been applied to discover and explain the developmental pathology of hypertension. According to previous animal studies, a variety of environmental stimuli received *in utero* appear to participate in the etiology of hypertension, including maternal overnutrition, maternal undernutrition, maternal renal insufficiency (3, 4), and others. The DOHaD concept posits that *in utero* stimuli influence offspring development and health by modifying the “epigenic phenotype” and forming an “epi-memory.” Among the major epigenetic modifications of DNA methylation, histone modification, and non-coding RNA, DNA methylation variance has garnered the most research attention to date (5). Epigenetic modifications are considered to be reversible, since they depend on the binding and release of chemical residues to DNA sequences and histone bonds. Therefore, using a balanced diet as a therapeutic agent immediately after birth may reverse the adverse effects caused by maternal malnutrition.

Studies on overnutrition suggest that high fructose intake during pregnancy can cause hypertension in offspring (6), and that excess fructose, fat, and salt intake have a synergistic effect on increased blood pressure in adult offspring (7, 8). Moreover, mothers fed a high-protein/low-carbohydrate diet during pregnancy tend to have offspring with increased blood pressure and a higher risk of glomerulosclerosis (9). Micronutrient deficiencies during pregnancy have also been linked to an increased risk of hypertension in offspring. For example, causal links have been demonstrated in maternal nutritional status characterized by deficits in calcium (10), iron (11), zinc (12), and vitamin D (13) with hypertension in the offspring. Besides the above programming insults, maternal protein restriction has been established as a common developmental origin of hypertension. Studies in rodents have consistently reported that 6–9% protein restriction in pregnant mothers resulted in hypertension in adult offspring (14–17). Previous studies led to several hypotheses about the underlying mechanisms, involving the suppression of the newborn renin-angiotensin system (18), impairment to nephrogenesis (19), triggering oxidative disruption in the medulla oblongata (20), or hindering the hypothalamic–pituitary–adrenal axis (21). However, there have been sparse reports on uncovering the role of epigenetic regulation in the pathology of hypertension.

We previously reported that maternal protein restriction modulates the methylation state of the renal prostaglandin E receptor 1 gene (*Ptger1*), a key regulator of hypertension, in the stroke-prone spontaneously hypertensive (SHRSP) rat model (17). In the present study, we administered SHRSP pups diets with different amounts of protein after a state of fetal protein restriction to investigate whether postnatal protein supplementation could rectify the negative changes in DNA methylation under malnutrition in pregnancy.

## 2. Materials and methods

### 2.1. Animal experiments

SHRSP rats obtained from Japan SLC, Inc. (Shizuoka, Japan) were kept under a 12 h light–dark cycle (light period 8:00–20:00), temperature of 22 ± 1°C, and humidity of 60 ± 5%. The animal experiments were approved and conducted in strict accordance with the guidelines stipulated by the Animal Usage Committee of the Graduate School of Agricultural and Life Sciences, University of Tokyo (approval no. P09-376).

**Figure 1:**
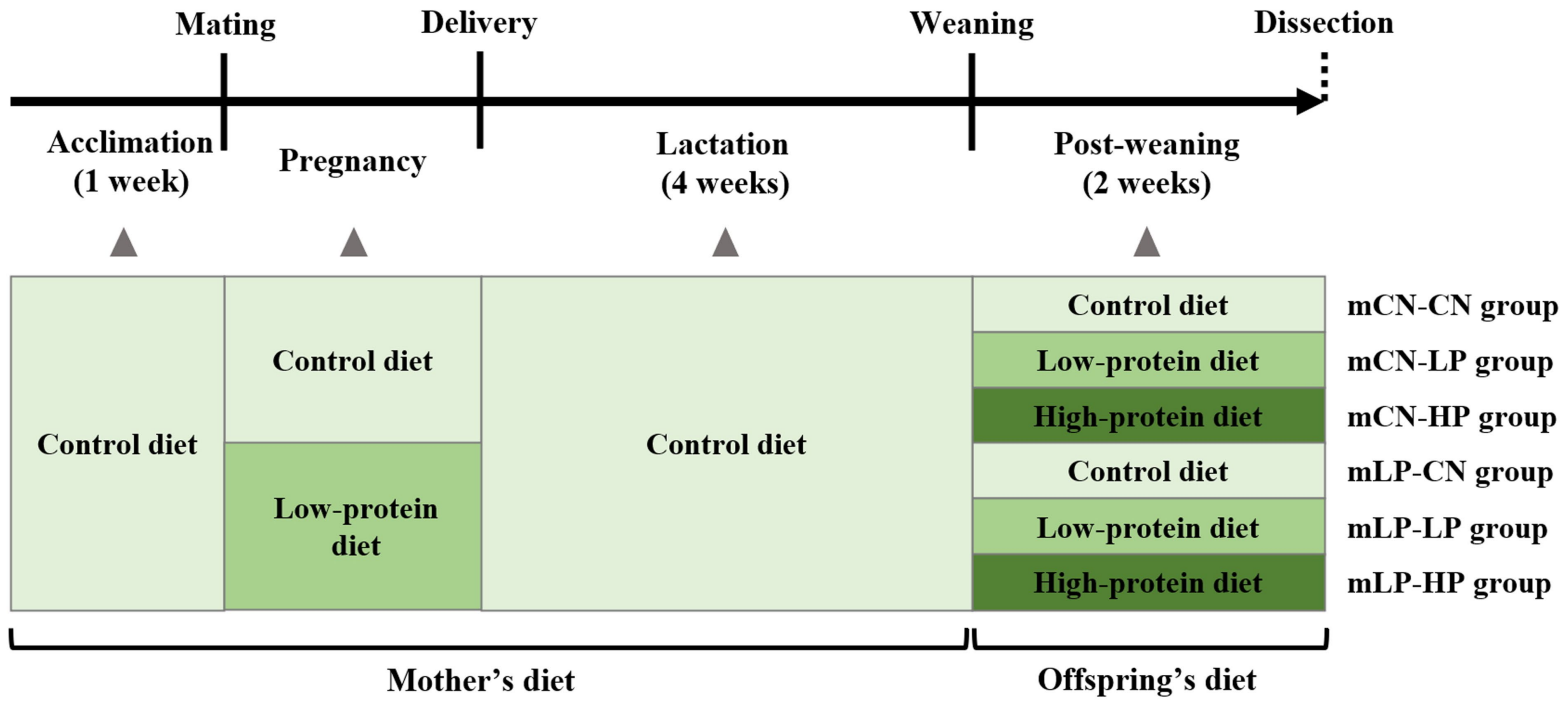
Schedule of the animal experiment. mLP-CN, maternal low-protein diet and control diet in offspring; mCN-CN, maternal control diet and control diet in offspring; mLP-LP, maternal low-protein diet and low-protein diet in offspring; mLP-HP, maternal low-protein diet and high-protein diet in offspring.

Nine-week-old male and female SHRSP rats were placed in separate cages for 1 week of acclimatization and fed a control (CN) diet as the normal diet. After acclimatization, male and female rats were mated by cohabiting overnight, and pregnancy was determined by the presence of a semen plug the following morning (day 0 of pregnancy). Pregnant dams were divided into two groups: those fed a CN diet *ad libitum* (mCN group) and those fed a low-protein diet (9% casein diet) *ad libitum* (mLP group). After delivery, all dams and offspring were fed the CN diet. At 28 days after birth, male pups were separated from the dams and divided into six groups (*n* = 5 per group) based on the pregnant dam’s diet and the diet of the pup. Pups in the mCN-CN and mLP-CN groups were fed the CN diet, those in the mCN-LP and mLP-LP groups were fed the low-protein diet, and those in the mCN-HP and mLP-HP groups were fed a high-protein diet (40% casein diet), each for 2 weeks. At six-weeks of age, male offspring were sacrificed, and their livers, kidneys, and intraperitoneal fat were collected. After weighing, a portion of the collected kidneys was cut for RNA extraction, soaked in RNA Later (Life Technologies Japan Ltd., Tokyo, Japan) at 4°C overnight, and then stored at −20°C. The remaining kidneys were immediately frozen in liquid nitrogen and stored at −80°C until analysis. The feed composition is shown in Supplementary Table S1 and the rearing schedule is shown in Figure 1. The body weights, total food intake, and tissue weights of the rats are shown in Supplementary Table S2.

### 2.2. DNA extraction

DNA from the kidney was extracted using DNAiso Reagent (Takara Bio, Tokyo, Japan). Briefly, 1.5 ml DNAiso Reagent was added to 40 mg of kidney tissue, crushed using a homogenizer, and left to stand for 5 min at room temperature. DNA was extracted from the homogenate according to the manufacturer’s protocol. The DNA quality was checked by electrophoresis (100 V, 20 min) using 1.2% agarose gel, and the DNA concentration was measured using a NanoDrop instrument (ND-1000, NanoDrop Technologies). The extracted DNA was stored at −80°C until analysis.

### 2.3. DNA methylation array

DNA methylation status was comprehensively analyzed by combining methylated DNA immunoprecipitation (MeDIP) and microarray technology outsourced to the MeDIP-chip microarray contract analysis service (Arraystar, Rockville, MD, USA). Briefly, 1 μg DNA of each sample was incubated for 10 min at 98°C with 1 optical density (OD) of Cy5-9mer primer (immunoprecipitation sample) or Cy3-9mer primer (input sample). Then, 100 pmol of deoxynucleoside triphosphates and 100 U of the Klenow fragment (New England Biolabs, Ipswich, MA, USA) were added and the mixture was incubated at 37°C for 2 h. The reaction was stopped by adding 0.1 volume of 0.5 M ethylenediaminetetraacetic acid and the labeled DNA was purified by isopropanol/ethanol precipitation. Microarrays were hybridized at 42°C for 16–20 h with Cy3/5-labeled DNA in NimbleGen hybridization buffer/hybridization component A in a hybridization chamber (Hybridization System, NimbleGen Systems, Inc., Madison, WI, USA). Following hybridization, washing was performed using the NimbleGen Wash Buffer kit (NimbleGen Systems, Inc.). For array hybridization, ArrayStar Rat RefSeq Promoter Array was used, which is a single-array design that includes 23,148 gene promoter regions (from approximately −1,300 bp to +500 bp of the transcription start sites) covered by approximately 180,000 probes with approximately 210 bp spacing, depending on the sequence composition of the region. For data normalization, to avoid technical variability and effectively evaluate methylation differences between samples, the raw data value was normalized using the log2-ratio. Median-centering, quantile normalization, and linear smoothing were performed by the Bioconductor packages Ringo, limma, and MEDME. The log2-normalized data were established for each sample and used in further peak-finding analysis. A sliding window (1,500 bp) peak-finding algorithm provided by NimbleScan v2.5 (Roche-NimbleGen) was applied to analyze the MeDIP-chip data. A one-sided Kolmogorov–Smirnov (KS) test was applied to determine whether the probes were drawn from a significantly more positive distribution of intensity log2-ratios than those in the rest of the array. Each probe was given a –log10 value of *p* score from the windowed KS test around that probe. If several adjacent probes rise significantly above a set threshold, the region is assigned to an enrichment peak. The peak data files were generated from the value of *p* data files. NimbleScan detects peaks by searching for at least two probes above a value of *p* minimum cutoff (−log10) of 2. Peaks within 500 bp of each other were merged.

### 2.4. Statistical analysis

Body weight, tissue weight, and food intake of the rats are expressed as the mean ± standard error. Multiple-comparison tests with two factors such as fetal protein nutrition and postnatal protein nutrition were performed using two-way analysis of variance and Tukey’s *post-hoc* test. Statistical significance was set at *p* < 0.05.

## 3. Results

### 3.1. Identification of differentially methylated CpG sites in the promoter regions

The methylation of DNA promoter regions affects the transcriptional expression of their downstream genes. Therefore, differentially methylated regions (DMRs) in the promoters were examined using DNA methylation arrays. First, the number of DMRs in each group was compared. Promoter regions were classified into three groups (high-CpG-density promoter, HCP; low-CpG-density promoter, LCP; and intermediate-CpG-density promoter, ICP) according to the CpG ratio, GC content, and length of the CpG-rich regions. HCPs are promoters containing a 500 bp interval from −0.7 kb to +0.2 kb with a (G + C) fraction ≥0.55 and a CpG observed-to-expected ratio (O/E) ≥ 0.6. An LCP is a promoter containing no 500 bp interval and a CpG O/E ≥ 0.4. ICPs are promoters that are neither HCPs nor LCPs. Compared with the mCN-CN group, 478 DMRs were hypermethylated and 576 were hypomethylated in the mLP-CN group (Table 1; Supplementary Figure S1). Compared to the mLP-CN group, 697 DMRs were hypermethylated and 405 were hypomethylated in the mLP-LP group, while 927 hypermethylated DMRs and 366 hypomethylated DMRs were obtained in the mLP-HP group vs. the mLP-CN group (Table 1; Supplementary Figure S1).

**Table 1:** Number of differentially methylated CpG sites of promoter regions in the kidney for comparisons between treatment groups.

Comparison	All regions	HCP	ICP	LCP
mLP-CN vs. mCN-CN				
Hypermethylated DMRs	478	280	129	69
Hypomethylated DMRs	576	264	152	160
Total DMRs	1,054	544	281	229
mLP-LP vs. mLP-CN				
Hypermethylated DMRs	697	408	172	117
Hypomethylated DMRs	405	188	124	93
Total DMRs	1,102	596	296	210
mLP-HP vs. mLP-CN				
Hypermethylated DMRs	927	486	233	208
Hypomethylated DMRs	366	162	107	97
Total DMRs	1,293	648	340	305

DMRs were classified into three categories—high-CpG-density promoter (HCP), low-CpG-density promoter (LCP), and intermediate-CpG-density promoter (ICP)—according to the CpG ratio, GC content, and length of CpG-rich regions. DMR, differentially methylated gene region; mLP-CN, maternal low-protein diet and control diet in offspring; mCN-CN, maternal control diet and control diet in offspring; mLP-LP, maternal low-protein diet and low-protein diet in offspring; mLP-HP, maternal low-protein diet and high-protein diet in offspring.

### 3.2. Pathway analysis of genes in the vicinity of DMRs altered by maternal protein restriction

**Figure 2:**
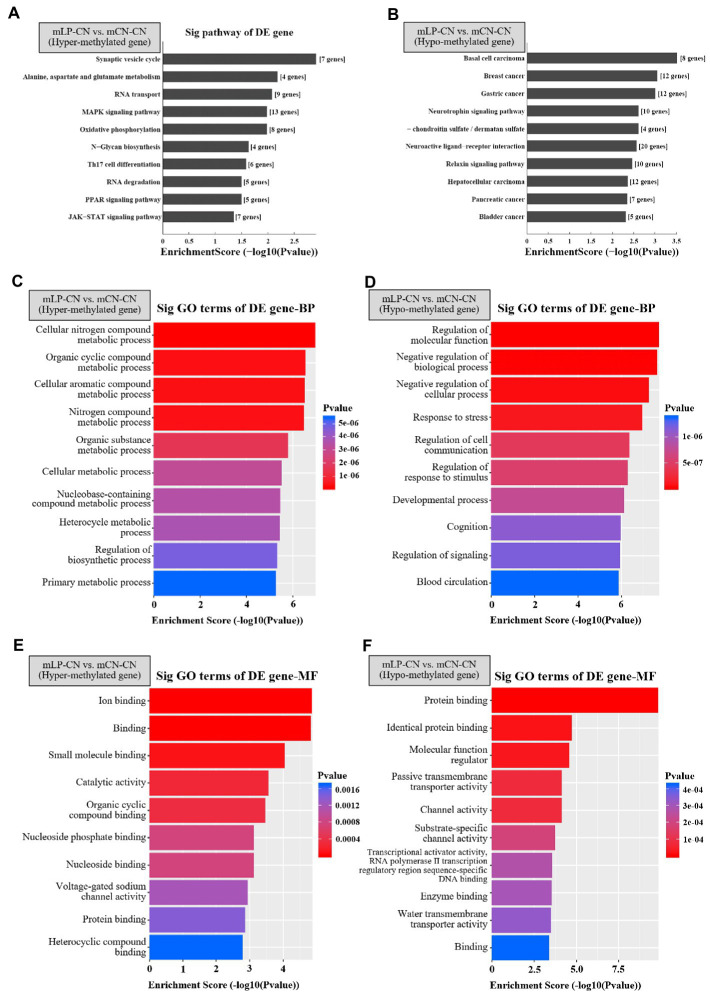
Top 10 signaling pathways of differentially expressed genes detected by methylation arrays in the kidney. Top pathways enriched in **(A)** hypermethylated or **(B)** hypomethylated genes in the mLP-CN group compared to the mCN-CN group are shown. Gene ontology of differentially expressed hypermethylated genes due to maternal low-protein intake for **(C)** biological process (BP) and **(E)** molecular function (MF) terms. Gene ontology of differentially expressed hypermethylated genes due to maternal low protein intake for **(D)** BP and **(F)** MF terms. Ten top gene ontology terms are shown. *p* < 0.05 was considered significant and –log10(*p* value) was calculated as the Enrichment Score. Sig, signaling; DE, differential expression; GO, gene ontology; BP, biological process; MF, molecular function; mLP-CN, maternal low-protein diet and control diet in offspring; mCN-CN, maternal control diet and control diet in offspring.

In the comparison of mLP-CN vs. mCN-CN, the top five signaling pathways with highest enrichment scores for the hypermethylated genes were synaptic vesicle cycle; alanine, aspartate, and glutamate metabolism; RNA transport; mitogen-activated protein kinase (MAPK) signaling pathway; and oxidative phosphorylation (Figure 2A). As a counterpart, hypomethylated genes were enriched in basal cell carcinoma, breast cancer, gastric cancer, neurotrophin signaling pathway, and glycosaminoglycan biosynthesis (Figure 2B). Next, Gene Ontology (GO) analysis of enriched biological process and molecular function terms was performed. As shown in Figures 2C,D, at the biological process level, the identified hypermethylated genes were mainly associated with various metabolic processes, whereas the hypomethylated genes were mainly involved in processes related to cellular progress regulation. As shown in Figures 2E,F, at the molecular function level, hypermethylated genes were mainly involved in molecular binding functions and catalytic activity, whereas the hypomethylated genes were mainly involved in disturbed protein binding functions.

### 3.3. Effect of postnatal protein intake on genes with altered DNA methylation status due to maternal protein restriction

Focusing on the pathways described in the previous section that possibly affect renal function and gene expression regulation, we examined whether genes with altered DNA methylation status due to maternal protein restriction could be reset by postnatal dietary protein correction. Among the genes that were altered to a hypermethylated state due to low maternal protein intake, *Ddx3x* (encoding the ATP-dependent RNA helicase DDX3X), *Ivd* (encoding mitochondrial isovaleryl-CoA dehydrogenase), *Pcgf6* (encoding polycomb group RING finger protein 6), *S100g* (encoding protein S100-G), and *Xrcc2* (encoding the DNA repair protein XRCC2) were identified as genes whose methylation status was reset by both low- and high-protein diets after birth. In addition, 11 genes were reset with a low-protein diet after birth, including *Adora2b* (adenosine receptor A2b) and *Aplf* (aprataxin and PNK-like factor), whereas genes reset by only a high-protein diet after birth included *Ar* (androgen receptor) and *Csf3* (granulocyte colony-stimulating factor) (Table 2).

**Table 2:** DNA methylation status in the kidney for genes that were altered to a hyper- or hypo-methylated state due to low maternal protein intake and reset by differences in dietary protein intake after birth.

Pathway/Gene ontology term	Gene symbol	Name	Promoter_Classification	Chromosome	PeakDMvalue		
					mLP-CN/ mCN-CN	mLP-LP/ mLP-CN	mLP-HP/ mLP-CN
Reset by both low- and high-protein diets after birth (Hypermethylated state)							
Cellular nitrogen compound metabolic process (BP)/ion binding (MF)/transcription factor binding (MF)	*Ddx3x*	ATP-dependent RNA helicase DDX3X	HCP	chrX	0.12	−0.29	−0.43
Ion binding (MF)	*Ivd*	Isovaleryl-CoA dehydrogenase, mitochondrial	ICP	chr3	0.21	−0.17	−0.54
Cellular nitrogen compound metabolic process (BP)/ion binding (MF)	*Pcgf6*	Polycomb group RING finger protein 6	HCP	chr1	0.28	−0.28	−0.36
ion binding (MF)	*S100g*	Protein S100-G	LCP	chrX	0.21	−0.44	−0.85
Cellular nitrogen compound metabolic process (BP)/ion binding (MF)	*Xrcc2*	DNA repair protein XRCC2	HCP	chr4	0.27	−0.42	−0.46
Reset by only low-protein diets after birth (Hypermethylated state)							
Cellular nitrogen compound metabolic process (BP)	*Adora* *2B*	Adenosine receptor A2b	HCP	chr10	0.34	−0.18	–
Cellular nitrogen compound metabolic process (BP)	*Aplf*	Aprataxin and PNK-like factor	ICP	chr4	0.22	−0.38	–
Cellular nitrogen compound metabolic process (BP)/ion binding (MF)	*Ddx10*	probable ATP-dependent RNA helicase DDX10	HCP	chr8	0.25	−0.31	–
Ion binding (MF)	*Gem*	GTP binding protein	HCP	chr5	0.46	−0.10	–
Cellular nitrogen compound metabolic process (BP)	*Hoxb7*	Homeobox protein Hox-B7	ICP	chr10	0.38	−0.47	–
ion binding (MF)	*Isca1*	Iron–sulfur cluster assembly 1 homolog	HCP	chr17	0.20	−0.28	–
MAPK signaling/cellular nitrogen compound metabolic process (BP)/ion binding (MF)/transcription factor binding (MF)	*Mapk* *14*	Mitogen-activated protein kinase 14	HCP	chr20	0.33	−0.16	–
Cellular nitrogen compound metabolic process (BP)/transcription factor binding (MF)	*Nbn*	Nibrin	HCP	chr5	0.27	−0.16	–
Oxidative phosphorylation/cellular nitrogen compound metabolic process (BP)	*Ndufa* *10*	NADH dehydrogenase [ubiquinone] 1 alpha	HCP	chr9	0.22	−0.21	–
Cellular nitrogen compound metabolic process (BP)	*Ndufa* *10 l1*	NADH dehydrogenase (ubiquinone) 1 alpha	HCP	chr9	0.22	−0.21	–
Ion binding (MF)	*Trpc5*	Short transient receptor potential channel 5	LCP	chrX	0.47	−0.31	–
Reset by only high-protein diets after birth (Hypermethylated state)							
Cellular nitrogen compound metabolic process (BP)/transcription factor binding (MF)	*Ar*	Androgen receptor	LCP	chrX	0.22	–	−0.36
JAK–STAT signaling/cellular nitrogen compound metabolic process (BP)	*Csf3*	Granulocyte colony-stimulating factor	LCP	chr10	0.26	–	−0.36
Reset by both low- and high-protein diets after birth (Hypomethylated state)							
Blood circulation (BP)/developmental process (BP)/response to stress (BP)	*Atp1b1*	Sodium/potassium-transporting ATPase subunit	HCP	ch13	−0.28	0.31	0.56
Regulation of signaling (BP)	*Ly6g6d*	Lymphocyte antigen 6 complex locus protein G6D	ICP	chr20	−0.28	0.15	0.28
Reset by only low-protein diets after birth (Hypomethylated state)							
Channel activity (MF)/developmental process (BP)/regulation of signaling (BP)/response to stress (BP)	*Bax*	Apoptosis regulator BAX	HCP	chr1	−0.27	0.43	–
Channel activity (MF)/regulation of signaling (BP)/response to stress (BP)	*Chrna4*	Neuronal acetylcholine receptor subunit alpha-4	HCP	chr3	−0.20	0.23	–
Developmental process (BP)/regulation of signaling (BP)	*Dll4*	Delta-like protein 4	ICP	chr3	−0.33	0.36	–
Developmental process (BP)/regulation of signaling (BP)/response to stress (BP)	*Lmna*	Prelamin-A/C isoform C2	ICP	chr2	−0.31	0.38	–
Developmental process (BP)	*Mafk*	Transcription factor MAFK	HCP	chr12	−0.24	0.26	–
Developmental process (BP)	*Neu1*	Sialidase-1 precursor	ICP	chr20	−0.38	0.22	–
Developmental process (BP)/response to stress (BP)	*Nfatc2*	Nuclear factor of activated T-cells, cytoplasmic	LCP	chr3	−0.39	0.47	–
Developmental process (BP)/response to stress (BP)	*Plg*	Plasminogen	LCP	chr1	−0.31	0.26	–
Developmental process (BP)/regulation of signaling (BP)/response to stress (BP)	*Vgf*	Neurosecretory protein VGF precursor	HCP	chr12	−0.33	0.35	–
Developmental process (BP)/regulation of signaling (BP)/response to stress (BP)	*Wnt5b*	Wingless-related MMTV integration site5B	LCP	chr4	−0.28	0.16	–
Reset by only high-protein diets after birth (Hypomethylated state)							
Blood circulation (BP)/developmental process (BP)/regulation of signaling (BP)	*Casq2*	Calsequestrin-2 precursor	LCP	chr2	−0.31	–	0.44
Developmental process (BP)	*Eif5a*	Eukaryotic translation initiation factor 5A-1	HCP	chr10	−0.31	–	0.38
Developmental process (BP)/regulation of signaling (BP)/response to stress (BP)	*Mapt*	Microtubule-associated protein tau	HCP	chr10	0.24	–	0.36
Developmental process (BP)	*Sp7*	Transcription factor SP7 isoform 2	LCP	chr7	0.30	–	0.52

mLP-CN, maternal low-protein diet and control diet in offspring; mCN-CN, maternal control diet and control diet in offspring; mLP-LP, maternal low-protein diet and low-protein diet in offspring; mLP-HP, maternal low-protein diet and high-protein diet in offspring; HCP, high-CpG-density promoter; LCP, low-CpG-density promoter; ICP, intermediate-CpG-density promoter. PeakDMvalue is the median log2-ratio from probes within the peak. A positive PeakDMvalue means high methylation and a negative value means low methylation. The area indicated by “–” represents no variation. For the genes in the table, the PeakDMvalue of the mLP-LP and mLP-HP groups did not vary compared to the mCN-CN group (data not shown).

In contrast, among the genes altered to a hypomethylated state due to maternal low-protein intake, *Atp1b1* (sodium/potassium-transporting ATPase subunit) and *Ly6g6d* (lymphocyte antigen 6 complex locus protein G6d) were identified to have a reset methylation status by both low- and high-protein diets after birth (Table 2). There were 10 genes reset by only a low-protein diet after birth (Table 2), including *Bax* (encoding the apoptosis regulator BAX) and *Chrna4* (encoding the neuronal acetylcholine receptor subunit alpha-4), whereas the genes reset by only a high-protein diet after birth included *Casq2* (calsequestrin-2 precursor), *Eif5a* (eukaryotic translation initiation factor 5A-1), *Mapt* (microtubule-associated protein tau), and *Sp7* (the transcription factor Sp7 isoform 2) (Table 2). For these genes whose hyper- or hypomethylated status was reset, the PeakDMvalue of the mLP-LP and mLP-HP groups did not vary compared to the mCN-CN group (data not shown).

## 4. Discussion

Maternal protein restriction hampers the health of offspring, causing long-lasting impacts by manipulating the descendent epigenome. In the present study, we investigated whether postnatal protein intake could remedy this situation at the methylation level.

We administered three diets to the pups of SHRSP rats under a state of dietary protein restriction during pregnancy: a CN diet with an “adequate” protein content, which is equivalent to the control condition; a low-protein diet to mimic a “continued restricted” protein content condition, representing a state of intergenerational undernutrition; and a high-protein diet to validate whether there could be an “excess compensation” effect. We therefore conducted three comparisons using methylome analyses, and found that postnatal CN remedy after maternal protein restriction still resulted in 1,054 DMRs compared with the state of non-protein deficiency throughout the study (mLP-CN vs. mCN-CN); however, cross-generation protein deficiency (mLP-LP) resulted in 1,102 DMRs in mLP-LP compared to the mLP-CN condition. Excessive protein also resulted in an altered methylated profile in the offspring. These results demonstrated that DNA methylation status in renal tissue is influenced by not only maternal nutrition but also postnatal nutritional status.

The methylated states of several genes that play important roles in renal disease and blood pressure regulation were found to be reset by postnatal low- or high-protein intake. Among them, postnatal high-protein administration reset the methylated states of *Atp1b1*, encoding an Na^+^/K^+^-ATPase as an important factor for blood pressure regulation because it reabsorbs sodium in the renal tubules (22–24). Polymorphisms of *Atp1b1* have been associated with blood pressure (22). The postnatal high-protein condition also reset the methylation status of *Xrcc2*, a key gene in the DNA repair process (25), and *Ar*, which plays a role in renal sodium and calcium excretion and in maintaining blood pressure (26). As AR is closely related to blood pressure regulation and has been reported to affect Enac-α expression (27, 28), it is possible that the *Ar* gene was hypermethylated upon low maternal protein intake and reset by a high-protein diet after birth may influence blood pressure in the offspring. For *Atp1b1* and *Xrcc2*, methylation was also reset by postnatal low-protein intake. This result is contrary to our expectation, and the mechanism of methylation resetting by both postnatal low- and high-protein intake should be investigated in the future. Interestingly, *Adora2b*, which mediates renal AMP-activated protein kinase (AMPK) activation (29), and *Trpc5*, whose abnormal expression was suggested to interfere with progressive kidney diseases (30), were subsequently altered at the methylation level due to postnatal protein restriction. Signaling pathways involving the adenosine receptor encoded by *Adora2b* have been reported to exert protective effects during acute kidney injury by inhibiting neutrophil-dependent tumor necrosis factor-alpha release (31). In addition, among the transient receptor potential (TRP) channels, short transient receptor potential channel 5 (TRPC5) has been identified as a cause of erythropoietin-induced hypertension in patients with chronic kidney disease (32). Therefore, alterations in DNA methylation and the reprogramming of this gene promoter region may play an important role in blood pressure regulation. The above genes may be crucial in the reprogramming progress. Thus, further studies using gene-editing techniques such as epigenome editing mice are encouraged to validate this hypothesis and explore the detailed mechanisms.

The top pathways enriched with the hypermethylated DMRs upon maternal protein restriction that may affect renal function included the MAPK, oxidative phosphorylation, PPAR, and JAK–STAT signaling pathways. RNA transport and degradation, which may affect gene expression, were also identified as top pathways. Although the specific activated/inhibited status of the indicated pathways requires further investigation, some of the factors involved in transcription or translation that affect associated gene expression may contain epigenetic markers. GO enrichment analysis revealed that the hypermethylated genes were mainly associated with voltage-gated sodium channel activity. This is consistent with previous studies demonstrating that *Ptger1*, a gene related to sodium retention in the kidney, can be reprogrammed by postnatal nutritional interventions (17). Conversely, the results of pathway and GO enrichment analyses showed that many hypomethylated DMRs due to maternal protein restriction were related to cancer, with only a few related to renal function. However, genes related to blood circulation, channel activity, and transcriptional activator activity, along with RNA polymerase II transcription regulatory region sequence-specific genes, may be associated with renal disease and gene expression regulation.

Several studies have suggested that an inappropriate nutritional environment for the mother during pregnancy increases the risk of disease development in the offspring. Our previous studies showed that offspring born to mothers who ingested a low-protein diet during pregnancy had an increased risk of salt-sensitive hypertension after birth, which was also associated with a shorter lifespan (33, 34). This result suggests that pregnant women should be aware of the importance of consuming an appropriate amount of protein during pregnancy. However, although pregnant women and their families need to obtain proper nutritional guidance, excessive interference with an expectant mother is not recommended, because pregnancy is physically and mentally demanding, food preferences and dietary intake change compared to pre-pregnancy states, and medication and supplementation during pregnancy are difficult. In addition, knowledge of appropriate nutrition during pregnancy is insufficient to prevent and treat people born with a predisposition to developing a disease. Although the approach suggested in many previous studies is to ameliorate the risk of disease in the offspring through improving the nutritional environment during pregnancy and lactation, we could propose that the postnatal nutritional environment is a novel and reasonable approach to prevent salt-sensitive hypertension.

A limitation of this study is that individual changes in expression at the gene and protein levels were not examined as we focused on DNA methylation. In addition, we did not examine whether the changes in DNA methylation status revealed in this study affect the development of salt-sensitive hypertension. Investigation of these changes would lead to a more accurate understanding of the epigenetic effects of the pregnancy and postnatal nutritional environment on offspring. Moreover, while a comprehensive examination using DNA methylation arrays was conducted, further examination of the DMRs considered in this analysis should be conducted through other methods, such as pyrosequencing. Since our previous study reported that maternal protein restriction could increase salt-sensitive blood pressure in male offspring (33, 34), we used only male offspring in the present study to elucidate the mechanism. However, future studies should also examine this mechanism in female offspring.

## 5. Conclusion

In this study, we showed that postnatal dietary protein supplementation may contribute to the reprogramming of an abnormal DNA methylation status caused by maternal malnutrition. Our results provide potential epigenetic targets for the treatment and prevention of hypertension caused by low maternal protein intake and offer a foundation for prevention strategies involving postnatal protein feeding.

## Data availability statement

The datasets presented in this study can be found in online repositories. The names of the repository/repositories and accession number(s) can be found at: https://www.ncbi.nlm.nih.gov/geo/query/acc.cgi?acc=GSE215182.

## Ethics statement

The animal study was reviewed and approved by the Animal Usage Committee of the Graduate School of Agricultural and Life Sciences, University of Tokyo.

## Author contributions

HK, HJ, and MM: conceptualization. MM and HJ: methodology. MM, XL, and KF: formal analysis. CA and SM: writing—original draft preparation. HJ and HK: writing—review and editing. All authors have read and agreed to the published version of the manuscript.

## Funding

This work was funded in part by a Grant-in-Aid (17H03815) from the Japan Society for the Promotion of Science.

## Conflict of interest

The authors declare that the research was conducted in the absence of any commercial or financial relationships that could be construed as a potential conflict of interest.

## Publisher’s note

All claims expressed in this article are solely those of the authors and do not necessarily represent those of their affiliated organizations, or those of the publisher, the editors and the reviewers. Any product that may be evaluated in this article, or claim that may be made by its manufacturer, is not guaranteed or endorsed by the publisher.
