# Supplementary material for: Postnatal nutrition environment reprograms renal DNA methylation patterns in offspring of maternal protein-restricted stroke-prone spontaneously hypertensive rats
Source: Front Nutr. 2023 Apr 13;10:1134955. doi: 10.3389/fnut.2023.1134955 (PMC10133489; doi:10.3389/fnut.2023.1134955)
Supplement: Supplementary file 1 [file Data_Sheet_1.docx]

Supplementary Material

# DNA Methylation Array Methods

## Immunoprecipitation

Sonicated genomic DNA (1 μg) was used for immunoprecipitation with a mouse monoclonal anti-5-methylcytosine antibody (Diagenode). The DNA was heat-denatured at 94 °C for 10 min, rapidly cooled on ice, and immunoprecipitated with 1 μL primary antibody overnight at 4 °C with rocking agitation in 400 μL immunoprecipitation buffer (0.5% bovine serum albumin in phosphate-buffered saline). To recover the immunoprecipitated DNA fragments, 200 μL of anti-mouse IgG magnetic beads were added and incubated for an additional 2 hours at 4 °C with agitation. After immunoprecipitation, a total of five immunoprecipitation washes were performed with ice-cold immunoprecipitation buffer. Washed beads were resuspended in TE buffer with 0.25% sodium dodecyl sulfate and 0.25 mg/mL proteinase K for 2 h at 65 °C and then allowed to cool down to room temperature. MeDIP DNA was purified using Qiagen MinElute columns (Qiagen).

## Whole- Genome Amplification (WGA)

The MeDIP-enriched DNA was amplified using a WGA kit from Sigma-Aldrich (GenomePlex® Complete Whole Genome Amplification (WGA2) kit). The amplified DNA samples were then purified with QIAquick PCR purification kit (Qiagen). The purified DNA was quantified using a NanoDrop ND-1000 spectrophotometer. For DNA labelling, the NimbleGen Dual-Color DNA Labeling Kit was used according to the manufacturer’s guidelines detailed in the NimbleGen MeDIP-chip protocol (NimbleGen Systems, Inc., Madison, WI, USA).

# Supplementary Figures and Tables


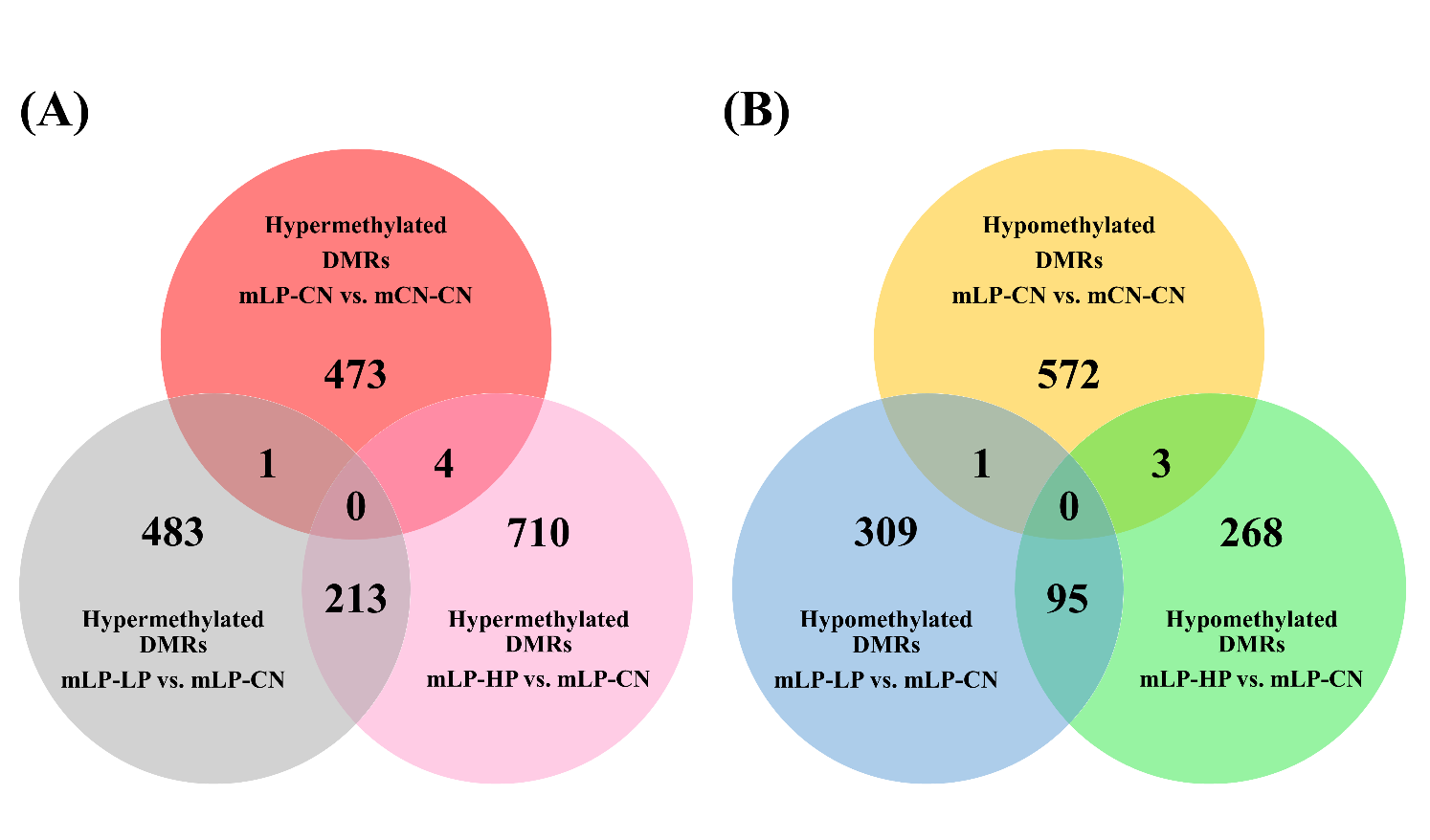


**Supplementary Figure S1.** Venn diagrams of the number differentially methylated CpG sites of promoter regions in the kidney for comparisons between treatment groups. (A) shows the number of hypermethylated DMRs (mLP-CN vs. mCN-CN) in the red circle, hypermethylated DMRs (mLP-LP vs. mLP-CN) in the gray circle and hypermethylated DMRs (mLP-HP vs. mLP-CN) in the pink circle. (B) shows the number of hypomethylated DMRs (mLP-CN vs. mCN-CN) in the yellow circle, hypomethylated DMRs (mLP-LP vs. mLP-CN) in the blue circle and hypomethylated DMRs (mLP-HP vs. mLP-CN) in the green circle.

Abbreviations: DMR, differentially methylated gene region; mLP-CN, maternal low-protein diet and control diet in offspring; mCN-CN, maternal control diet and control diet in offspring; mLP-LP, maternal low-protein diet and low-protein diet in offspring; mLP-HP, maternal low-protein diet and high-protein diet in offspring.

**Supplementary Table S1.** Feed composition.

| **Component (%)** | **Control diet**  **(20% Casein)** | **Low-protein diet**  **(9% Casein)** | **High-protein diet**  **(40% Casein)** |
| --- | --- | --- | --- |
| Casein | 20.0 | 9.0 | 40.0 |
| Corn starch | 66.8 | 77.8 | 46.8 |
| dl-Methionine | 0.2 | 0.2 | 0.2 |
| Soybean oil | 5.0 | 5.0 | 5.0 |
| Vitamin mixture * | 1.0 | 1.0 | 1.0 |
| Mineral mixture * | 4.0 | 4.0 | 4.0 |
| Cellulose powder | 3.0 | 3.0 | 3.0 |

*AIN-76 prescription (Oriental Yeast Co., Ltd., Tokyo, Japan).

**Supplementary Table S2. (A)** Final body weight of 6-week-old rat pups; weight increase from 4 to 6 weeks of age; total food intake from 4 to 6 weeks of age; tissue weights of the liver, kidney, and abdominal fat. **(B)** Results of two-way ANOVA. For total food intake calculation, the total weight of the food box and the food within it was measured, and the difference reduction from the previous measurement was calculated as the amount of food intake. Measurements were performed twice per week, and the total for the two weeks was calculated as the total food intake.

**(A)**

| **Group** | **Body weight**  **(g)** | **Weight increase (g)** | **Total food intake (g)** | **Liver**  **(g)** | **Kidney**  **(g)** | **Abdominal fat (g)** |
| --- | --- | --- | --- | --- | --- | --- |
| **mCN-CN** | 132.7 | 72.3 ^a^ | 172.5 | 5.8 ^ab^ | 1.3 ^a^ | 2.2 |
| **mCN-LP** | 113.0 | 56.6 ^b^ | 168.3 | 4.4 ^a^ | 1.1 ^b^ | 2.3 |
| **mCN-HP** | 130.5 | 73.0 ^a^ | 168.8 | 6.2 ^b^ | 1.6 ^c^* | 2.1 |
| **mLP-CN** | 117.2 | 66.0 | 158.1 | 4.7 | 1.2 ^de^ | 1.8 |
| **mLP-LP** | 113.1 | 57.2 | 168.5 | 4.2 | 1.0 ^d^ | 2.3 |
| **mLP-HP** | 117.0 | 67.2 | 153.5 | 5.4 | 1.4 ^e^* | 1.8 |

**(B)**

| **Factor** | **Body weight** | | **Weight increase** | | **Total food intake** | | **Liver** | | **Kidney** | | **Abdominal fat** | |
| --- | --- | --- | --- | --- | --- | --- | --- | --- | --- | --- | --- | --- |
|  | **F value** | **P value** | **F value** | **P value** | **F value** | **P value** | **F value** | **P value** | **F value** | **P value** | **F value** | **P value** |
| **in maternal diet** | 3.627 | 0.069 | 2.469 | 0.129 | 2.550 | 0.123 | 4.909 | 0.036 | 6.512 | 0.018* | 2.016 | 0.169 |
| **in offspring diet** | 2.248 | 0.127 | 12.393 | < 0.001** | 0.474 | 0.628 | 8.145 | 0.002** | 20.383 | < 0.001** | 1.943 | 0.165 |
| **interaction** | 0.954 | 0.399 | 0.837 | 0.445 | 0.668 | 0.522 | 0.634 | 0.539 | 1.017 | 0.377 | 0.393 | 0.679 |

(A) Values are expressed as the mean ± standard error (*n* = 5). Statistical analysis was performed using two‑way ANOVA and Tukey’s *post-hoc* test. Values are expressed as the mean ± standard error (*n* = 5). Statistical significance was set at *p* < 0.05. Different letters (a, b, and c) indicate statistically significant differences (in the mCN groups). Different letters (d and c) indicate statistically significant differences (in the mLP groups). * indicates statistically significant differences between the mCN-HP and mLP-HP groups.

Abbreviations: mLP-CN, maternal low-protein diet and control diet in offspring; mCN-CN, maternal control diet and control diet in offspring; mLP-LP, maternal low-protein diet and low-protein diet in offspring; mLP-HP, maternal low-protein diet and high-protein diet in offspring; HCP, high-CpG-density promoter; LCP, low-CpG-density promoter; ICP, intermediate-CpG-density promoter.

(B) **p* < 0.05, ***p* < 0.001.

**Supplementary Table S3.** DNA microarray from the kidney. **(A–C)** Genes that were altered to a hypermethylated state due to low maternal protein intake and reset by differences in protein intake after birth. **(D–F)** Genes that were altered to a hypomethylated state due to low maternal protein intake that were reset by differences in protein intake after birth. **(A)** and **(D)** show the genes that were reset by both low- and high-protein diets after birth, **(B)** and **(E)** show the genes were reset by only low-protein diets after birth, and **(C)** and **(F)** show the genes that were reset by only high-protein diets after birth.

**(A)**

| **Pathway/Gene Ontology term** | **Gene  symbol** | **Name** | **ID** | **Log fold change** | | |
| --- | --- | --- | --- | --- | --- | --- |
|  |  |  |  | **LP-CN/ mCN-CN** | **mLP-LP/ mLP-CN** | **mLP-HP/ mLP-CN** |
| Cellular nitrogen compound metabolic process (BP)/ion binding (MF)/transcription factor binding (MF) | *Ddx3x* | ATP-dependent RNA helicase DDX3X | 1372242_at | 0.12 | 0.14 | –0.04 |
|  |  |  | 1386535_at | 1.11 | 0.08 | –1.17 |
| Ion binding (MF) | *Ivd* | isovaleryl-CoA dehydrogenase, mitochondrial | 1370232_at | 0.04 | –0.21 | –0.18 |
| Cellular nitrogen compound metabolic process (BP)/ion binding (MF) | *Pcgf6* | polycomb group RING finger protein 6 | 1389760_at | 0.10 | 0.02 | –0.18 |
| Ion binding (MF) | *S100g* | protein S100-G | 1368339_at | –0.84 | 0.18 | 0.26 |
| Cellular nitrogen compound metabolic process (BP)/ion binding (MF) | *Xrcc2* | DNA repair protein XRCC2 | 1393405_at | –0.10 | 0.22 | –0.34 |

**(B)**

| **Pathway/Gene Ontology term** | **Gene  symbol** | **Name** | **ID** | **Log fold change** | | |
| --- | --- | --- | --- | --- | --- | --- |
|  |  |  |  | **LP-CN/ mCN-CN** | **mLP-LP/ mLP-CN** | **mLP-HP/ mLP-CN** |
| Cellular nitrogen compound metabolic process (BP) | *Adora2b* | adenosine receptor A2b | 1387395_at | –0.15 | –0.15 | –0.16 |
| Cellular nitrogen compound metabolic process (BP) | *Aplf* | aprataxin and PNK-like factor | 1373994_at | 0.36 | –0.10 | –0.67 |
| Cellular nitrogen compound metabolic process (BP)/ion binding (MF) | *Ddx10* | probable ATP-dependent RNA helicase DDX10 | 1373549_at | 0.04 | 0.12 | 0.09 |
| Ion binding (MF) | *Gem* | GTP binding protein | 1382351_at | 0.69 | –0.34 | –0.16 |
| Cellular nitrogen compound metabolic process (BP) | *Hoxb7* | homeobox protein Hox-B7 | 1388287_at | 0.07 | 0.06 | –0.03 |
| Ion binding (MF) | *Isca1* | iron-sulfur cluster assembly 1 homolog | 1388603_at | 0.18 | –0.05 | 0.14 |
|  |  |  | 1388613_at | 0.19 | –0.06 | 0.05 |
| MAPK signaling/cellular nitrogen compound metabolic process (BP)/ion binding (MF)/transcription factor binding (MF) | *Mapk14* | mitogen activated protein kinase 14 | 1367697_at | 0.14 | 0.05 | –0.37 |
| Cellular nitrogen compound metabolic process (BP)/transcription factor binding (MF) | *Nbn* | nibrin | 1387977_at | 0.20 | 0.06 | –0.19 |
| Oxidative phosphorylation/cellular nitrogen compound metabolic process (BP) | *Ndufa10* | NADH dehydrogenase [ubiquinone] 1 alpha | 1397206_at | 0.17 | –0.04 | –0.28 |
| Cellular nitrogen compound metabolic process (BP) | *Ndufa10l1* | NADH dehydrogenase (ubiquinone) 1 alpha | 1389334_at | –0.34 | 0.08 | 0.14 |
| Ion binding (MF) | *Trpc5* | short transient receptor potential channel 5 | 1369368_at | –0.20 | 0.21 | 0.29 |

**(C)**

| **Pathway/Gene Ontology term** | **Gene  symbol** | **Name** | **ID** | **Log fold change** | | |
| --- | --- | --- | --- | --- | --- | --- |
|  |  |  |  | **LP-CN/ mCN-CN** | **mLP-LP/ mLP-CN** | **mLP-HP/ mLP-CN** |
| Cellular nitrogen compound metabolic process (BP)/transcription factor binding (MF) | *Ar* | androgen receptor | 1369159_at | -0.39 | 0.04 | –0.22 |
| JAK-STAT signaling/cellular nitrogen compound metabolic process (BP) | *Csf3* | granulocyte colony-stimulating factor | 1369529_at | 0.03 | –0.09 | 0.07 |

**(D)**

| **Pathway/Gene Ontology term** | **Gene  symbol** | **Name** | **ID** | **Log fold change** | | |
| --- | --- | --- | --- | --- | --- | --- |
|  |  |  |  | **LP-CN/ mCN-CN** | **mLP-LP/ mLP-CN** | **mLP-HP/ mLP-CN** |
| Blood circulation (BP)/developmental process (BP)/response to stress (BP) | *Atp1b1* | sodium / potassium-transporting ATPase subunit | 1367814_at | 0.18 | –0.07 | –0.14 |
|  |  |  | 1386937_at | 0.20 | –0.02 | 0.07 |
| Regulation of signaling (BP) | *Ly6g6d* | lymphocyte antigen 6 complex locus protein G6d | - | ND | ND | ND |

**(E)**

| **Pathway/Gene Ontology term** | **Gene  symbol** | **Name** | **ID** | **Log fold change** | | |
| --- | --- | --- | --- | --- | --- | --- |
|  |  |  |  | **LP-CN/ mCN-CN** | **mLP-LP/ mLP-CN** | **mLP-HP/ mLP-CN** |
| Channel activity (MF)/developmental process (BP)/regulation of signaling (BP)/response to stress (BP) | *Bax* | apoptosis regulator BAX | 1369122_at | –0.05 | –0.01 | –0.25 |
| Channel activity (MF)/regulation of signaling (BP)/response to stress (BP) | *Chrna4* | neuronal acetylcholine receptor subunit alpha-4 | 1369252_at | –0.14 | 0.02 | 0.09 |
|  |  |  | 1391490_at | 0.30 | 0.02 | –0.13 |
| Developmental process (BP)/regulation of signaling (BP) | *Dll4* | delta-like protein 4 | 1379790_at | –0.24 | 0.26 | 0.06 |
|  |  |  | 1394367_at | 0.08 | 0.16 | –0.25 |
| Developmental process (BP)/regulation of signaling (BP)/response to stress (BP) | *Lmna* | prelamin-A/C isoform C2 | 1368054_at | –0.08 | -0.03 | –0.24 |
|  |  |  | 1368055_at | –0.03 | –0.10 | –0.03 |
| Developmental process (BP) | *Mafk* | transcription factor MafK | 1372211_at | –0.02 | 0.14 | 0.01 |
| Developmental process (BP) | *Neu1* | sialidase-1 precursor | 1369081_at | 0.58 | –0.11 | –0.27 |
|  |  |  | 1375684_at | 0.27 | –0.15 | –0.15 |
| Developmental process (BP)/response to stress (BP) | *Nfatc2* | nuclear factor of activated T-cells, cytoplasmic | 1381185_at | –0.26 | –0.02 | 0.34 |
| Developmental process (BP)/response to stress (BP) | *Plg* | plasminogen | 1368360_at | –0.28 | –0.05 | 0.57 |
| Developmental process (BP)/regulation of signaling (BP)/response to stress (BP) | *Vgf* | neurosecretory protein VGF precursor | 1368359_at | –0.08 | 0.09 | 0.10 |
| Developmental process (BP)/regulation of signaling (BP)/response to stress (BP) | *Wnt5b* | wingless-related MMTV integration site5B | 1374970_at | –0.01 | 0.06 | 0.04 |

**(F)**

| **Pathway/Gene Ontology term** | **Gene  symbol** | **Name** | **ID** | **Log fold change** | | |
| --- | --- | --- | --- | --- | --- | --- |
|  |  |  |  | **LP-CN/ mCN-CN** | **mLP-LP/ mLP-CN** | **mLP-HP/ mLP-CN** |
| Blood circulation (BP)/developmental process (BP)/regulation of signaling (BP) | *Casq2* | calsequestrin-2 precursor | 1368988_at | –0.09 | 0.00 | –0.21 |
|  |  |  | 1387401_at | –0.23 | 0.02 | 0.32 |
| Developmental process (BP) | *EifI5a* | eukaryotic translation initiation factor 5A-1 | 1371329_at | 0.18 | -0.07 | -0.15 |
| Developmental process (BP)/regulation of signaling (BP)/response to stress (BP) | *Mapt* | microtubule-associated protein tau | 1368137_at | –0.01 | -0.07 | 0.04 |
|  |  |  | 1398138_at | –0.31 | -0.06 | 0.02 |
|  |  |  | 1387071_at | 0.04 | -0.11 | –0.19 |
| Developmental process (BP) | *Sp7* | transcription factor SP7 isoform 2 | - | ND | ND | ND |

Abbreviations: BP, biological process; MF, molecular function; ND, not determined; mLP-CN, maternal low-protein diet and control diet in offspring; mCN-CN, maternal control diet and control diet in offspring; mLP-LP, maternal low-protein diet and low-protein diet in offspring; mLP-HP, maternal low-protein diet and high-protein diet in offspring; HCP, high-CpG-density promoter; LCP, low-CpG-density promoter; ICP, intermediate-CpG-density promoter.

Log fold change is the median log2-ratio from probes within the peak. A positive value indicates increased gene expression and a negative value indicates decreased gene expression. The area indicated by “-” indicates no variation.
